# Dietary Antioxidants and Redox Signaling in Cancer Prevention: Mechanistic Insights and Metabolic Inflammation

**DOI:** 10.3390/nu18101552

**Published:** 2026-05-14

**Authors:** Viorel Ispas, Viviana Maggio, Hibo Said Hassan, Asya Ilayda Sayilgan, Faadumaqamar Mahamed Hassan, Sorina Ispas, Manfredi Rizzo

**Affiliations:** 1 Department of Anatomy, Faculty of General Medicine, “Ovidius” University, 900470 Constanta, Romania; viorel.ispas@365.univ-ovidius.ro (V.I.);; 2 Department of Health Promotion Sciences Maternal and Infantile Care, Internal Medicine and Medicinal Specialties (PROMISE), School of Medicine, University of Palermo, 90133 Palermo, Italy; 3 RAK College of Pharmacy, RAK Medical and Health Sciences University, Ras Al Khaimah 11172, United Arab Emirates

**Keywords:** dietary antioxidants, oxidative stress, reactive oxygen species, redox homeostasis, cancer prevention, gut microbiota, Nrf2 signaling, inflammasome, metabolic inflammation

## Abstract

Oxidative stress is an important component of cancer biology and is characterized by an imbalance between the production of reactive oxygen species (ROS) and antioxidant defense systems. Excess ROS can cause molecular damage and genomic instability; at the same time, ROS signaling remains necessary for normal cellular function. Redox homeostasis is of particular importance in this balance. The aim of this structured narrative review was to summarize and critically discuss current evidence on how dietary antioxidants influence redox-sensitive pathways involved in cancer prevention, with particular attention to metabolic inflammation, mitochondrial quality control, and gut microbiota-related mechanisms. We performed a structured literature search of Scopus, Web of Science, and PubMed, focusing on articles published between 2021 and 2026. The evidence covered major redox-sensitive pathways, including Nrf2-Keap1-ARE signaling, AMPK-mTOR regulation, NF-κB-mediated inflammation, mitochondrial quality control (autophagy and mitophagy), and inflammasome activation. These pathways, which are involved in tumor initiation and progression, link oxidative stress to metabolic and inflammatory processes. Current evidence suggests that dietary antioxidants act primarily by supporting endogenous defense systems. This may help explain the “antioxidant paradox”, in which antioxidant-rich dietary patterns are associated with a lower risk of cancer. In some studies, high-dose supplementation with isolated antioxidants has produced inconsistent or sometimes adverse results. These effects depend on dose, chemical form, metabolic context, and baseline redox state. The gut microbiota is also an important mediator of antioxidant bioactivity; by converting dietary polyphenols into bioactive metabolites, it can influence systemic redox balance and metabolic signaling. This microbiota-dependent modulation may partially explain inter-individual variability in responses to dietary interventions. In conclusion, dietary antioxidants should be considered as modulators of redox-sensitive signaling networks, not merely as simple radical scavengers. Personalized modulation of redox homeostasis is a future strategy for cancer prevention, with a greater emphasis on whole-diet and biomarker-guided approaches.

## 1. Introduction

Oxidative stress reflects a dynamic imbalance between reactive oxygen species (ROS) generation and antioxidant defense systems [1]. Excessive ROS promote DNA damage and tumor initiation [2]. Tightly controlled ROS signaling is essential for physiological cellular processes and immune responses. This dual role underscores that redox homeostasis is not a static equilibrium but a finely regulated system that determines whether oxidative signals support adaptation or drive pathological transformation [3]. ROS exert context-dependent effects in carcinogenesis [4], and multiple factors influence this context—for example, ROS concentration and metabolic state. Moderate and sustained ROS levels activate redox-sensitive signaling pathways such as MAPK/ERK and PI3K/Akt, promoting tumor growth and survival [5,6], whereas excessive ROS accumulation induces oxidative damage and triggers cell death mechanisms, including apoptosis and ferroptosis [7]. Cancer cells adapt to this oxidative environment by upregulating endogenous antioxidant systems, e.g., the Nrf2-Keap1 axis, allowing them to maintain ROS at levels that support proliferation while avoiding cytotoxicity [8]. This adaptive redox regulation represents a critical challenge in developing effective prevention and therapeutic strategies [9]. Dietary antioxidants are considered to be protective against cancer through their ability to neutralize ROS [10]. This antioxidant paradox highlights the complexity of redox homeostasis [11]. Randomized trials using high-dose isolated antioxidant supplements have reported inconsistent or even adverse outcomes [12]. Dietary antioxidants may influence redox-sensitive signaling pathways beyond direct ROS scavenging [13].

This structured narrative review aims to summarize the available evidence on the role of dietary antioxidants related to redox signaling in cancer prevention. We focus on key molecular pathways, metabolic and inflammatory crosstalk, and microbiota-mediated effects, while emphasizing the context-dependent nature of antioxidant interventions and their implications for personalized, evidence-based prevention strategies. Throughout this review, we distinguish prevention and early carcinogenesis from established tumor biology, because redox pathways may have opposite implications across these stages.

## 2. Materials and Methods

### 2.1. Review Design and Reporting Framework

This structured narrative review was designed to summarize and interpret the available evidence on the molecular, metabolic, and translational roles of dietary antioxidants in the modulation of oxidative stress relevant to cancer prevention. Because this is a structured narrative review, the methodological transparency and reporting quality of the review were guided by the Scale for the Assessment of Narrative Review Articles (SANRA), which emphasizes the importance of a clear rationale, explicit aims, transparent literature-search description, appropriate referencing, scientific reasoning, and clear presentation of evidence [14]. 

This review was not designed as a systematic review or scoping review. No PRISMA flow diagram, formal risk-of-bias assessment, or quantitative synthesis was performed. 

### 2.2. Search Strategy

A structured literature search was performed in PubMed, Scopus, and Web of Science.

The search focused primarily on articles published between 2021 and 2026, and the last search was conducted on 27 March 2026. The search was limited to articles in English. 

Search terms included combinations of keywords and database-specific indexing terms related to “dietary antioxidants”, “phytochemicals”, “polyphenols”, “oxidative stress”, “reactive oxygen species”, “redox signaling”, “redox homeostasis”, “cancer prevention”, “early carcinogenesis”, “Nrf2 signaling”, “NF-κB”, “AMPK”, “mTOR”, “inflammasome”, “NLRP3”, “autophagy”, “mitophagy”, “gut microbiota”, and “metabolic inflammation”. The full database-specific search strategy and applied filters are provided in Appendix A (Table A1).

Earlier seminal studies, landmark randomized trials, large cohort studies, and key mechanistic papers published before 2021 were also included when they were directly relevant to the conceptual framework of the review, particularly for antioxidant supplementation, Mediterranean dietary patterns, redox signaling, and cancer prevention outcomes.

### 2.3. Selection Criteria

Eligible publications included human, animal, experimental, translational, clinical, observational, systematic review, meta-analysis, and narrative review articles addressing dietary antioxidants, phytochemicals, antioxidant-rich dietary patterns, oxidative stress, redox biology, metabolic inflammation, mitochondrial quality control, gut microbiota-mediated metabolism, or cancer prevention.

Studies were considered to be eligible when they contributed to one or more of the main thematic domains of the review: redox-sensitive signaling pathways, Nrf2-Keap1-ARE signaling, AMPK-mTOR regulation, NF-κB-mediated inflammation, inflammasome activation, autophagy and mitophagy, metabolic inflammation, gut microbiota-mediated biotransformation, whole-food dietary patterns, or isolated antioxidant supplementation.

Conference abstracts without full text, editorials, opinion pieces, letters, case reports without mechanistic discussion, non-English publications, and studies without clear relevance to dietary antioxidants, redox biology, or cancer prevention were excluded. The inclusion and exclusion criteria are summarized in Appendix A (Table A2).

Two authors independently screened the retrieved literature for relevance to the objectives of this structured narrative review. Disagreements were resolved through discussion and, when necessary, consultation with a senior author.

### 2.4. Evidence Synthesis

The included literature was synthesized narratively and organized by theme. The synthesis focused on molecular mechanisms of oxidative stress modulation; dietary antioxidants and phytochemicals as redox-modulating bioactives; interactions between metabolic, inflammatory, mitochondrial, and gut microbiota-related pathways; and translational evidence from dietary-pattern studies and antioxidant supplementation trials.

Because the evidence base included mechanistic, preclinical, translational, observational, and clinical studies, particular attention was given to distinguishing mechanistic plausibility from direct human cancer-prevention evidence. Where appropriate, this manuscript explicitly differentiates cancer prevention and early carcinogenesis from established tumor biology, since redox-sensitive pathways may have different implications depending on disease stage.

## 3. Redox Homeostasis and Reactive Oxygen Species (ROS) Signaling in Cancer Development

### 3.1. Oxidative Stress as a Contemporary Concept in Cancer Prevention

Reactive oxygen species (ROS) exert paradoxical roles in cancer. At moderately elevated levels, they promote tumor growth and angiogenesis through activation of redox-sensitive signaling pathways [15]. When ROS levels exceed antioxidant defenses, they cause cytotoxic damage and trigger cell death [7]. Cancer cells adapt their antioxidant defenses to survive chronic oxidative stress [9]. At the same time, they exploit ROS to support invasion and metastasis. Redox homeostasis represents an important determinant at every stage of tumorigenesis [16]. 

ROS can activate important signaling pathways, including MAPK/ERK and PI3K/Akt, to support cellular proliferation [17]. Excessive ROS can induce oxidative damage to proteins, nucleic acids, and lipids when antioxidant defenses are overwhelmed [18].

Antioxidant supplementation may also exert context-dependent effects in cancer [15]. In certain oncogene-driven tumors, antioxidant supplementation can promote tumor progression [19]. For example, in KRAS-driven lung cancer, antioxidant supplementation may accelerate tumor progression [20], while in BRAF-driven melanoma it can accelerate metastasis by reducing oxidative stress [21].

High ROS levels trigger genomic instability through mutagenic DNA lesions such as 8-oxo-dG [18]. ROS also promote epithelial-to-mesenchymal transition (EMT), which underlies metastatic spread [16].

Oxidative stress is associated with hallmarks of cancer, driving metabolic reprogramming [22] and chronic inflammation [23]. This altered redox homeostasis creates vulnerabilities in tumor cells that can be therapeutically exploited [15]. Excessive ROS induce oxidative cell death pathways, including ferroptosis and pyroptosis [7].

### 3.2. Physiological ROS Signaling Versus Pathological Oxidative Stress

At physiological levels, ROS participate in regulated redox signaling required for normal cellular function—a concept often referred to as oxidative eustress [24]. These molecules include free radicals (e.g., superoxide anions) and non-radicals (e.g., hydrogen peroxide). Signaling mainly occurs through redox switches, regulating important pathways that control cell-cycle regulation, differentiation, proliferation, and immune responses. Maintaining redox homeostasis is a dynamic challenge, often described as “homeodynamics” [3].

Spatiotemporal control of ROS is important for maintaining redox homeostasis. Hydrogen peroxide (H_2_O_2_) concentrations differ by orders of magnitude between organelles. Redox signaling follows principles that regulate the spatiotemporal dynamics of cellular processes. Specific aquaporins facilitate transmembrane diffusion of H_2_O_2_, enabling its function as a controlled intracellular messenger [17].

When ROS generation exceeds antioxidant defenses, “oxidative distress” occurs. This imbalance causes severe oxidative damage to proteins, lipids, and nucleic acids, leading to cell death through apoptosis or necrosis [25].

At low-to-moderate levels, ROS stimulate tumorigenesis by inducing genetic mutations and promoting proliferation through signaling pathways [16]. Cancer cells often amplify their antioxidant systems, such as the Nrf2 pathway, to limit ROS to levels that promote growth while avoiding cytotoxicity [26]. Metabolic reprogramming, including the Warburg effect, contributes to redox homeostasis by modulating mitochondrial function and ROS production [27].

**Figure 1:**
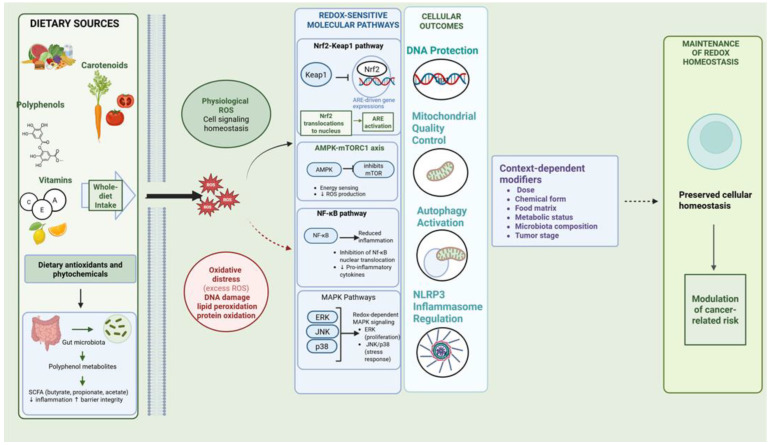
Dietary antioxidants and redox signaling in cancer prevention. Solid arrows indicate direct pathway relationships, dashed arrows indicate indirect or modulatory relationships, and colored circles indicate representative cellular or molecular processes. Created in BioRender. Ispas, S. (2026) https://BioRender.com/fsw3l5u, accessed on 13 May 2026. Created in BioRender. Ispas, S. (2026) https://BioRender.com/fsw3l5u, accessed on 13 May 2026.

Figure 1 summarizes the main redox-sensitive mechanisms through which dietary antioxidants and phytochemicals may influence cancer prevention.

Dietary antioxidants and phytochemicals modulate redox homeostasis through multiple molecular pathways, including Nrf2-Keap1 signaling, AMPK-mTORC1 regulation, NF-κB-mediated inflammation, and MAPK pathways. These coordinated mechanisms support mitochondrial quality control, autophagy, and NLRP3 inflammasome regulation, ultimately contributing to cellular homeostasis and cancer risk modulation.

### 3.3. Inflammasome–ROS Crosstalk in Cancer Prevention

Inflammasomes are multiprotein complexes that detect cellular danger signals and regulate immune and inflammatory responses. The interaction between inflammasomes and ROS is bidirectional and context-dependent, contributing to tumor-promoting and tumor-suppressive effects [28]. The NLRP3 inflammasome is the most studied member of the NLR family; it requires a two-step activation mechanism [29]: First, a “priming” signal induces the expression of inflammasome components through NF-κB activation. Then, an activating signal triggers assembly of the protein complex. Mitochondrial ROS acts as an activator signal, linking oxidative stress to inflammatory responses [30].

In normal cells and early malignant transformation, inflammasome activation facilitates the elimination of damaged cells through pyroptosis [29]. This process limits the accumulation of oncogenic mutations and contributes to immune surveillance and tissue homeostasis [31]. Experimental studies show that inflammasome deficiency increases cancer susceptibility in multiple animal models [32].

Chronic inflammasome-mediated inflammation can create a tumor microenvironment that favors cancer progression [31]. This process influences multiple stages of tumor development. Persistent activation of the NLRP3 inflammasome leads to the release of pro-inflammatory cytokines, e.g., IL-1β and IL-18 [29], which promote tumor angiogenesis and extracellular matrix remodeling; it also recruits myeloid-derived suppressor cells (MDSCs) [31]. This dual role illustrates how timing and the cellular environment determine the function of the inflammasome in cancer.

Multiple control mechanisms, such as mitochondrial autophagy (mitophagy) and endogenous antioxidant systems, regulate the interaction between ROS and inflammasomes [33]. IL-10, an anti-inflammatory cytokine, inhibits NLRP3 inflammasome activation in macrophages [31]. These regulatory processes suggest that the modulation of redox balance could influence inflammasome activity and the antitumor immune response. 

Dietary antioxidants can modulate inflammasome activation through several mechanisms, including the regulation of mitochondrial ROS and the modulation of inflammatory gene expression through Nrf2 signaling [34]; they also influence the polarization of macrophages toward pro- or anti-inflammatory phenotypes. The Dietary Antioxidant Index (DAI) quantifies the intake of vitamins A, C, and E, as well as minerals such as zinc and selenium. Studies show an inverse association between the DAI and chronic diseases, including cancer [35].

At physiological concentrations (50–200 µM), vitamin C functions as a conventional antioxidant, neutralizing ROS and maintaining redox balance. In contrast, pharmacological doses (millimolar range) can exert pro-oxidant effects by generating ascorbate radicals and hydrogen peroxide (H_2_O_2_) in the extracellular environment [36]. This shift in redox activity may contribute to cytotoxic effects in certain tumor contexts. These dose-dependent dual effects highlight the need for precise titration of antioxidant interventions.

## 4. Conceptual Framework

### 4.1. The Antioxidant Paradox in Cancer Prevention

The relationship between antioxidants and cancer prevention is often seen as a paradox [19]. This paradox arises from the dual nature of reactive oxygen species (ROS) [37]. Landmark randomized trials, including ATBC, CARET, and SELECT, reported no overall protective effect and, in selected populations, potential harm from high-dose isolated antioxidant supplementation [38,39,40]. These results reshaped the current understanding of the role of oxidative stress in cancer. 

Dietary patterns rich in antioxidants, such as the Mediterranean diet, have been associated with lower cancer risk in epidemiological studies [41]. These findings suggest that protective effects arise from interactions between many bioactive compounds, fiber, and other dietary components, rather than from individual antioxidants alone. 

### 4.2. Thresholds for Adaptive Signaling and Redox Hormesis

The concept of hormesis provides a framework for understanding the complex relationship between ROS and cellular health; it represents an adaptive response that enhances cellular resilience and protects against subsequent oxidative damage [42].

Mitohormesis is a specific form of redox hormesis involving mitochondria; it occurs when mild mitochondrial stress triggers adaptive responses. In mitohormesis, transient mitochondrial ROS production activates signaling pathways that increase cellular resistance to future metabolic and oxidative stress. Exercise induces transient increases in the production of mitochondrial ROS, which act as signaling molecules to promote adaptive cellular responses [43].

Conversely, excessive reduction in ROS levels in cancer cells through antioxidant supplementation may create conditions that favor tumor survival and growth, potentially enhancing tumor progression [19]. 

## 5. Mechanism-Based Classification of Dietary Antioxidants and Redox-Modulating Bioactives

In this review, the term “dietary antioxidants” is used as a broad nutritional term that includes food-derived antioxidant compounds, phytochemicals, and redox-modulating dietary bioactives. Some of these compounds may act through direct radical scavenging, whereas others primarily influence redox-sensitive signaling through hormetic responses, electrophilic stress, Nrf2-Keap1-ARE signaling, NF-κB modulation, AMPK activation, mitochondrial quality-control pathways, or gut microbiota-mediated biotransformation. Therefore, the term does not imply that all listed compounds act mainly through stoichiometric ROS scavenging.

Dietary antioxidants primarily modulate redox signaling [44]. 

Table 1 summarizes the main redox-sensitive molecular pathways and representative exposure contexts relevant to cancer prevention.

### 5.1. Polyphenols

Selected polyphenols and polyphenol-related dietary bioactive compounds, including resveratrol, curcumin, quercetin, epigallocatechin gallate (EGCG), and anthocyanin-derived metabolites, can regulate molecular signaling pathways and epigenetic mechanisms relevant to cancer prevention [64,65]. 

Resveratrol and curcumin activate the Nrf2 transcription factor [26]. Stabilized Nrf2 moves to the nucleus and binds antioxidant response elements (AREs), inducing the expression of cytoprotective genes such as HO-1, NQO-1, SOD, and catalase, which neutralize reactive oxygen species and prevent oxidative damage [66]. 

Polyphenols inhibit the NF-κB pathway, which is chronically activated during aging [51]. This decreases pro-inflammatory cytokines such as TNF-α, IL-6, and IL-1β, thereby limiting persistent inflammation. Polyphenols also activate AMP-activated protein kinase (AMPK), which is involved in the regulation of cellular metabolism. AMPK activation is associated with improved mitochondrial function, enhanced autophagy, and broader redox-adaptive signaling responses [44].

Polyphenols can modulate gene expression using epigenetic mechanisms [65]; they can inhibit DNA methyltransferases (DNMTs), reversing aberrant methylation of promoter regions—including the Nrf2 gene—to restore antioxidant gene expression [67]. Many polyphenols also inhibit histone deacetylases (HDACs), promoting histone acetylation and chromatin relaxation, and prevent transcriptional repression of tumor-suppressor and anti-inflammatory genes [64].

### 5.2. Carotenoids

High dietary intake of carotenoids from fruits and vegetables is associated with a lower risk of cancer, including breast cancer [68]. 

Large, randomized trials, including the Alpha-Tocopherol, Beta-Carotene Cancer Prevention (ATBC) Study and the Beta-Carotene and Retinol Efficacy Trial (CARET), were terminated early because high-dose β-carotene increased the incidence of lung cancer and mortality in smokers [38,39]. Lifestyle-based approaches suggest that the protective effects of antioxidants are more pronounced when derived from whole dietary patterns rather than isolated supplementation [69]. 

Under conditions of elevated oxidative stress, or at high concentrations, carotenoids can shift from protective antioxidants to pro-oxidant activity [70]. This context-dependent behavior explains why dietary carotenoids are beneficial, whereas high-dose supplementation can be harmful in certain populations.

### 5.3. Organosulfur Compounds and Electrophilic Phytochemicals

Keap1 functions as a redox sensor that regulates the activation of cytoprotective pathways; its cysteine residues detect oxidative stress and promote NRF2 activation, thereby inducing phase II detoxifying enzymes, which protect cells against carcinogens and harmful oxidants [45]. Sulforaphane (SFN) is an isothiocyanate and organosulfur compound derived from cruciferous vegetables; it modulates cell survival pathways and activates the Nrf2 signaling pathway, enhancing cellular antioxidant defenses [71].

Garlic compounds, such as diallyl disulfide (DADS) and diallyl trisulfide (DATS), exert anticancer effects through multiple molecular mechanisms, including induction of apoptosis and inhibition of cell proliferation [72]. 

### 5.4. Vitamins with Antioxidant Properties (C and E)

Isolated antioxidant vitamin supplementation has produced inconsistent and, in some cases, adverse outcomes in cancer prevention. High-dose vitamin E and C supplements have not demonstrated protective effects against tumor development [73]. In some studies, antioxidant supplementation was associated with an increased incidence of cancer. Large clinical trials report heterogeneous findings. The SELECT trial ended early when vitamin E raised the risk of prostate cancer [38].

Vitamin C exhibits dose-dependent biological effects. Oral supplementation is limited by intestinal absorption and homeostatic control of plasma levels. Supplements may be beneficial in patients with documented deficiencies. In individuals with adequate baseline levels, supplementation has shown no clear benefit and may even be harmful. Intravenous administration achieves millimolar plasma concentrations and exerts pro-oxidant cytotoxic effects in tumor cells. Chronic high-dose oral vitamin C may increase oxalate production and has been associated with an increased risk of kidney stone formation in some populations [36]. Current evidence supports prioritizing the prevention of deficiencies through diet rather than routine high-dose supplementation [69].

## 6. Molecular Mechanisms of Oxidative Stress Modulation

### 6.1. Nrf2-Keap1-ARE Axis

The Nrf2-Keap1-ARE axis is a major pathway that regulates the cellular response to oxidative stress. Under basal conditions, Nrf2 is sequestered in the cytoplasm by Keap1, which facilitates proteasomal degradation [46]. Under conditions of cellular stress or exposure to reactive species, Keap1 undergoes conformational modifications that lead to the release and stabilization of Nrf2. Stabilized Nrf2 accumulates in the cell and translocates to the nucleus. Once Nrf2 enters the nucleus, it binds to antioxidant response elements (AREs) located in the promoter regions of specific target genes. This binding initiates the transcription of cytoprotective genes involved in antioxidant defense and detoxification. This mechanism allows cells to respond to stress while maintaining a balanced redox state, rather than eliminating ROS. Once activated, Nrf2 induces an antioxidant network that includes phase II detoxification enzymes along with the glutathione and thioredoxin systems [34].

Phase II detoxifying enzymes contribute to the neutralization of harmful compounds and facilitate their elimination, while glutathione and thioredoxin systems maintain intracellular redox homeostasis. At the same time, these systems do not eliminate ROS. Instead, they restore the redox balance, which allows normal signaling to continue. This regulation of oxidative stress is an important mechanism through which dietary antioxidants and cellular defense systems maintain homeostasis [46].

In normal cells, during the early stages of carcinogenesis, Nrf2 protects cells by activating antioxidant defenses and facilitating the detoxification of carcinogens [45]. Nrf2 limits DNA damage and prevents the initiation of malignant transformation. In established tumors, persistent activation of Nrf2 may contribute to cancer progression. Genetic alterations in KEAP1, NFE2L2, or related pathways can lead to continuous Nrf2 activation, which enables cancer cells to maintain stronger antioxidant defenses, resist treatment, and adapt to metabolic stress [34].

Nrf2 is often called a “double-edged sword”, since it protects cells at early stages but can promote tumor growth in advanced cancer [74].

Nrf2 appears most relevant to cancer prevention in normal cells and during early carcinogenesis, where it may limit oxidative damage and support carcinogen detoxification [26].

In established tumors, however, persistent Nrf2 activation may also favor tumor survival and metabolic adaptation, which makes its role clearly stage-dependent [74].

Most of the available evidence comes from experimental and mechanistic studies, while direct evidence for cancer prevention in humans remains limited [46].

Overall, the evidence is predominantly mechanistic and preclinical; its directness to cancer prevention is biologically plausible but stage-dependent. The main limitation remains the limited availability of direct human prevention evidence.

### 6.2. AMPK-mTOR-IGF-1 Signaling

The AMPK-mTOR-IGF-1 axis links cellular energy sensing with redox and oxidative stress control [47]. AMPK is activated by elevated AMP/ATP ratios and functions as a sensor of cellular energy status. Concurrently, AMPK enhances antioxidant defenses through NAD^+^-dependent activation of the SIRT1/PGC-1α–FoxO pathway, enhancing the expression of antioxidant enzymes such as superoxide dismutases (SODs) and catalase. This shift from anabolic activity to stress adaptation helps cells maintain redox balance when nutrients are limited or when the cell is under oxidative stress [44]. Upon activation, AMPK suppresses anabolic pathways by inhibiting mTORC1, conserving energy [47], and modulating metabolic processes associated with ROS production [27]. 

In contrast, mTORC1 and IGF-1 signaling supports anabolic growth under nutrient-rich conditions and actively regulates cellular redox [49]. IGF-1 signaling is also implicated in antioxidant protection and redox regulation [75]. These mechanisms help to limit intracellular hydrogen peroxide accumulation. Even though this balance supports normal tissue growth, persistent activation of the PI3K-AKT-mTOR pathway can disturb the redox homeostasis. This disturbance increases oxidative stress, which may cause cell damage and dysfunction. This effect is often seen in hyperinsulinemia and metabolic syndrome. When this pathway is disrupted, redox imbalance may contribute to insulin resistance and the development of cancer. Long-term oxidative stress activates inhibitory enzymes such as PTEN and PTP1B, which impair IGF-1 and insulin signaling, contributing to metabolic dysfunction [44].

Reduced AMPK activity in obesity and diabetes fails to restrain mTORC1, thereby enhancing anabolic signaling and promoting mitochondrial dysfunction. In cancer, this signaling network plays context-dependent roles. AMPK can function as a tumor suppressor by restraining mTOR-driven biosynthesis and metabolic reprogramming [47]. Established tumors exploit AMPK-mediated metabolic adaptation and redox buffering to survive hypoxia, nutrient deprivation, and therapy-induced oxidative stress. Collectively, the AMPK-mTOR-IGF-1 axis links metabolic stress to redox control and cancer progression [76].

This pathway is likely to be most relevant to cancer prevention through its effects on metabolic regulation and early stress adaptation [47].

At the same time, much of the available evidence is mechanistic, and in established tumors AMPK-related signaling may also support survival under metabolic stress [76].

Direct evidence linking this pathway to cancer prevention outcomes in humans remains limited [48].

Overall, the evidence is predominantly mechanistic and translational; its directness to cancer prevention is mainly indirect, through metabolic regulation and early stress adaptation. The main limitation is the lack of prevention-focused human studies assessing this pathway as a modifiable target.

### 6.3. NF-κB and Redox-Sensitive Inflammatory Signaling

NF-κB functions as a key redox-sensitive transcription factor that couples oxidative stress to inflammatory signaling [52]. Reactive oxygen species (ROS) regulate multiple steps of the IKK-IκB-NF-κB cascade in a context-dependent manner: low-to-moderate levels promote pathway activation, whereas excessive ROS impair signaling through oxidative modification of pathway proteins [76]. Upon activation, NF-κB induces pro-inflammatory cytokines such as IL-6, TNF-α, and IL-1β, and it can also enhance the production of ROS-generating enzymes. This creates a self-amplifying inflammatory loop that reinforces redox imbalance and sustains chronic inflammatory signaling [50]. 

Within the tumor microenvironment, hypoxic and metabolically stressed conditions elevate mitochondrial and NOX-derived ROS production [76], promoting persistent NF-κB activation across tumor and stromal compartments [50]. This redox-driven signaling reprograms tumor-associated macrophages toward an immunosuppressive state and activates cancer-associated fibroblasts through NOX4-TGF-β, amplifying inflammatory and oxidative cues [16]. The resulting inflammatory environment advances angiogenesis, invasion, immune evasion, and therapy resistance. This makes ROS-dependent NF-κB signaling a key driver of tumor-promoting inflammation in stressed cells [76].

From a prevention perspective, NF-κB is most relevant in the setting of chronic inflammation and early carcinogenic change [23].

However, a large part of the available evidence comes from studies of tumor biology and the tumor microenvironment, so its direct relevance to cancer prevention in humans remains partially indirect [50].

Overall, the evidence is biologically strong but much less direct from a prevention-focused clinical perspective [52].

Overall, the evidence is predominantly mechanistic and translational; its directness to cancer prevention is indirect but biologically plausible in chronic inflammatory and early carcinogenic settings. The main limitation is the reliance on tumor-biology and tumor-microenvironment studies rather than prevention-focused human research.

### 6.4. Autophagy and Mitophagy as Redox Quality-Control Mechanisms

Autophagy and mitophagy act as central redox quality-control mechanisms by selectively eliminating damaged, ROS-producing mitochondria, thereby preserving mitochondrial network integrity [54]. This process limits oxidative damage to cellular macromolecules [53]. Redox stress engages autophagy and mitophagy via AMPK-mTOR-ULK1 signaling pathways [77]. In parallel, it induces mitophagy via the stabilization of PINK1 on depolarized mitochondria, which recruits Parkin for the clearance of damaged organelles [55]. 

Impaired function of mitophagy regulators, such as PINK1, Parkin, and BNIP3, leads to increased oxidative stress, promoting a shift toward glycolytic metabolism [54]. These alterations may contribute to cellular transformation and tumor progression. In established tumors, these quality-control processes are co-opted to support survival under hypoxia and nutrient deprivation. Autophagy and mitophagy exhibit stage-dependent dual roles in carcinogenesis, suppressing tumor initiation early while supporting metabolic adaptation and stress tolerance in advanced disease [53].

Autophagy and mitophagy are most plausibly linked to cancer prevention through the maintenance of mitochondrial integrity and the limitation of oxidative damage in early carcinogenesis [33].

Conversely, in established tumors, these same processes may support cellular survival under stress, which underscores their stage-dependent role [53].

Most of the available data come from experimental models, and direct preventive evidence in humans is still lacking [54].

Overall, the evidence is predominantly mechanistic and preclinical; its directness to cancer prevention is plausible in early carcinogenesis through mitochondrial quality control, but it remains stage-dependent. The main limitation is the lack of direct prevention-specific human evidence.

## 7. Inflammasome Activation as a Redox-Sensitive Link Between Oxidative Stress, Metabolism, and Cancer Risk

### 7.1. ROS as Upstream Activators of Inflammasomes

Mitochondrial reactive oxygen species (mtROS) act as early danger signals, linking mitochondrial damage to the activation of the NLRP3 inflammasome. This mechanism represents a pathway through which mitochondrial dysfunction promotes inflammasome assembly and inflammatory signaling. Excess mtROS promote opening of the mitochondrial permeability transition pore (mPTP) and voltage-dependent anion channel (VDAC) oligomerization, enabling cytosolic release of oxidized mitochondrial DNA, which serves as a damage-associated molecular pattern that directly engages and activates the NLRP3 inflammasome [78].

In addition to acute activation, ongoing chronic low-grade oxidative stress sustains a basal level of activity of the NLRP3 inflammasome, promoting a persistent pro-inflammatory state linked to cancer risk and prevention [79]. ROS contribute to inflammasome priming and activation. Mitochondrial reactive oxygen species and cytosolic mitochondrial DNA promote NF-κB activation through the cGAS-STING pathway to upregulate NLRP3 and pro-IL-1β [78]. At the same time, ongoing mitochondrial damage provides the second signal required for inflammasome assembly [30]. Continuous NLRP3 signaling advances a pro-tumorigenic microenvironment by increasing the production of inflammatory cytokines and angiogenic factors [79]; it also drives immunosuppressive programs and promotes epithelial transformation and tumor progression under chronic oxidative stress [56].

### 7.2. NLRP3 Inflammasome, Metabolic Inflammation, and Redox-Regulated Carcinogenesis

Metabolic inflammation associated with obesity and insulin resistance creates a chronic inflammatory environment for NLRP3 inflammasome activation, linked to increased cancer risk. NLRP3-mediated IL-1β impairs insulin signaling and reinforces metabolic dysfunction. Hypoxia amplifies inflammasome activation via HIF-1α-mediated metabolic reprogramming and ATP release. These mechanisms sustain low-grade systemic inflammation (“metaflammation”), promoting early carcinogenesis [57].

Lipotoxic mediators activate TLR4-NF-κB signaling and increase mitochondrial ROS. Macrophage-driven feedback loops sustain chronic inflammation [80].

Autophagy and mitophagy act as counter-regulatory mechanisms. These processes limit the accumulation of mitochondrial ROS and damage-associated molecular patterns [33]. Efficient mitophagy prevents excessive inflammasome activation and preserves cellular homeostasis [28].

Dietary antioxidants and phytochemicals may modulate NLRP3 inflammasome activity through upstream redox and mitochondrial pathways involved in priming and assembly [51].

Polyphenols such as curcumin and quercetin suppress NF-κB priming and reduce the expression of NLRP3 and pro-IL-1β levels. Other compounds, such as resveratrol, interfere with mitochondrial dynamics and endoplasmic reticulum–mitochondria contact sites, which are required for inflammasome assembly [51].

The relevance of inflammasome signaling to cancer prevention seems strongest in early inflammatory and metabolic dysregulation, where chronic low-grade inflammation may contribute to carcinogenesis [57].

Still, much of the current evidence is mechanistic, and the effects of inflammasome activation appear to depend on tissue context and disease stage [28].

Direct human evidence linking inflammasome modulation to cancer prevention outcomes remains limited [29].

Overall, the evidence is predominantly mechanistic and translational; its directness to cancer prevention is biologically plausible but context-dependent, particularly in obesity-associated metabolic inflammation and early inflammatory carcinogenesis. The main limitation is the limited availability of direct human evidence linking inflammasome modulation to cancer prevention outcomes.

## 8. Gut Microbiota as a Mediator of Antioxidant Bioactivity

**Figure 2:**
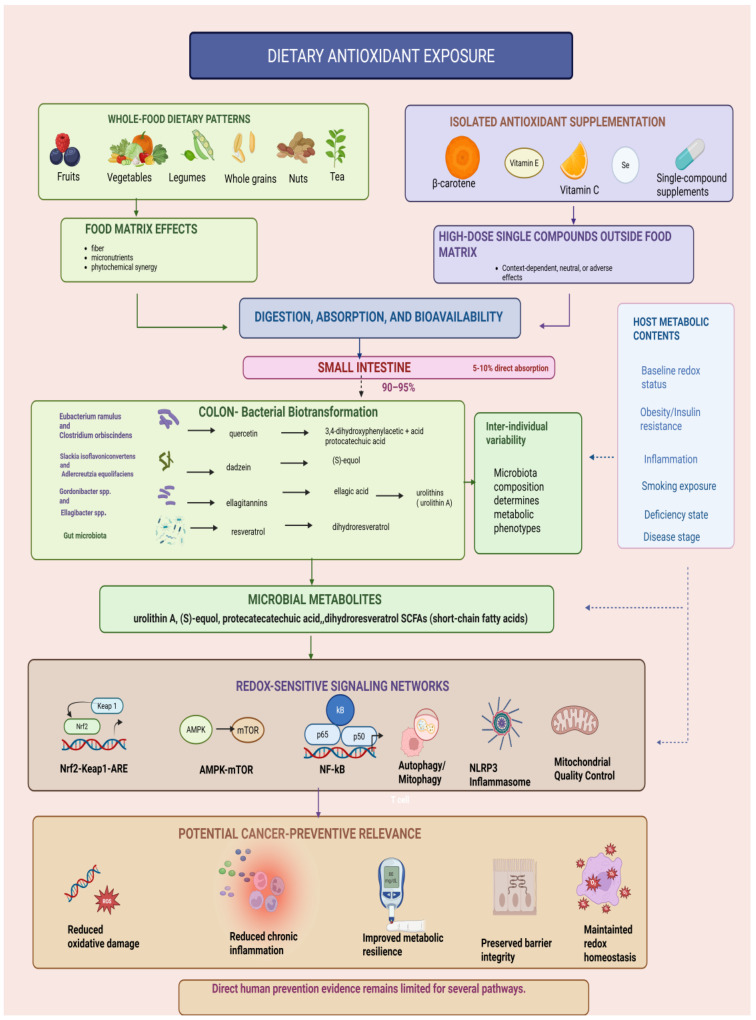
Conceptual framework linking dietary antioxidant exposure, host metabolic context, redox-sensitive signaling, and cancer-prevention relevance Solid arrows indicate the main proposed sequence from dietary exposure to bioavailability, microbial metabolism, redox-sensitive signaling, and potential cancer-preventive relevance. Dashed arrows indicate modulatory influences of host metabolic context and indirect feedback relationships. Created in BioRender. Ispas, S. (2026) https://BioRender.com/xc5fsn4, accessed on 13 May 2026.

To integrate the translational sequence from dietary exposure to biological response, Figure 2 presents a conceptual framework linking whole-food dietary patterns, food-matrix effects, isolated supplementation, bioavailability, microbiota-mediated metabolism, host metabolic context, redox-sensitive signaling networks, and potential cancer-preventive relevance.

Whole-food dietary patterns provide antioxidants and phytochemicals within complex food matrices. Isolated antioxidant supplementation delivers selected compounds outside the natural food matrix, and often at higher doses. Bioavailability, gut microbiota-mediated metabolism, baseline redox status, metabolic health, inflammation, smoking exposure, deficiency states, and disease stage may influence downstream effects on Nrf2-Keap1-ARE signaling, AMPK-mTOR regulation, NF-κB-mediated inflammation, autophagy/mitophagy, NLRP3 inflammasome activity, and mitochondrial quality control. These mechanisms may contribute to cancer-preventive relevance through the modulation of oxidative damage, inflammatory tone, metabolic resilience, barrier integrity, and redox homeostasis, although direct human prevention evidence remains limited for several pathways. Within this framework, gut microbiota-mediated biotransformation represents one important mechanism through which dietary polyphenols and other antioxidant-rich food components may generate bioactive metabolites such as equol, urolithins, and phenolic acids. Representative microbial mediators include *Eubacterium ramulus* and *Clostridium orbiscindens* in flavonoid metabolism, *Slackia isoflavoniconvertens* and *Adlercreutzia equolifaciens* in equol production, and *Gordonibacter* spp. and *Ellagibacter* spp. in urolithin formation. These microbiota-derived metabolites may influence intestinal barrier integrity, inflammatory signaling, oxidative stress responses, and redox-sensitive pathways, thereby contributing to inter-individual variability in the potential cancer-preventive effects of dietary antioxidants.

### 8.1. Parent Compounds Versus Microbial Metabolites

The bioavailability and systemic efficacy of dietary polyphenols depend on colonic biotransformation by gut microbiota. Only 5–10% of ingested polyphenols are absorbed in the small intestine as free compounds; 90–95% reach the colon, where resident microbes catalyze extensive structural modifications [58]. These transformations involve deglycosylation, C-ring cleavage, dehydroxylation, demethylation, and hydrogenation [59]. Mammalian enzymes lack the capacity to cleave the core flavonoid ring system, rendering microbial catabolism indispensable for enabling their biological activity [60].

Specific bacterial taxa orchestrate class-dependent transformations. Quercetin undergoes sequential deglycosylation and ring fission by *Eubacterium ramulus* and *Clostridium orbiscindens*, producing 3,4-dihydroxyphenylacetic acid and protocatechuic acid. These metabolites exhibit potent anti-inflammatory, neuroprotective, and insulin-sensitizing activities that are superior to those of the parent flavonol [61]. Similarly, daidzein is converted through a four-step enzymatic cascade by *Slackia isoflavoniconvertens* and *Adlercreutzia equolifaciens* into (S)-equol, an estrogenic metabolite with enhanced cardiovascular and bone-protective effects [60]. Ellagitannins, poorly absorbed in their native form, are hydrolyzed to ellagic acid and subsequently transformed by *Gordonibacter* spp. and *Ellagibacter* spp. into urolithins, particularly urolithin A [62].

The superior bioactivity of microbial metabolites extends to resveratrol, whose gut-derived dihydroresveratrol exhibits greater tissue retention and anti-proliferative capacity than resveratrol itself. Catechin- and anthocyanin-derived microbial metabolites modulate metabolic signaling by activating SIRT-1-mediated autophagy and improving insulin sensitivity [81,82]. Fermentation-induced deglycosylation exposes additional phenolic hydroxyl groups, enhancing radical scavenging capacity and lipid peroxidation inhibition beyond those of unfermented precursors [63]. These findings suggest that the biological effects attributed to polyphenol-rich foods may be substantially influenced by microbially generated metabolites. These effects vary according to polyphenol class, host absorption, phase II metabolism, tissue distribution, and gut microbiota composition. Direct human evidence linking microbiota-derived polyphenol metabolites to cancer prevention outcomes remains limited. Inter-individual variability in microbiota composition may determine metabolic phenotypes and therapeutic responses [58]. However, not all microbial species are beneficial in this context, and the biological effects of dietary polyphenols depend on the composition of the gut microbiota and the metabolic capacity of specific taxa [60,61].

The biological effects of dietary polyphenols depend on gut microbial biotransformation and on the presence and metabolic activity of specific bacterial taxa [60]. *Gordonibacter* spp. and *Ellagibacter* spp. have been associated with the generation of urolithins from ellagitannins [62]. These observations support the view that the gut microbiota is an important and variable mediator of antioxidant bioactivity [61].

Overall, the evidence is predominantly mechanistic, bioavailability-based, and translational; its directness to cancer prevention remains indirect through effects on microbial metabolism, redox signaling, inflammatory tone, barrier integrity, and inter-individual responses to polyphenol-rich diets. The main limitation is the scarcity of prevention-specific human studies linking defined microbial metabolites to cancer outcomes.

### 8.2. Intestinal Barrier Integrity and Ahr–Nrf2 Signaling

Microbiota-derived polyphenol metabolites exert protective effects on intestinal epithelial integrity through the modulation of tight junction architecture and redox-sensitive signaling pathways. These mechanisms reduce paracellular permeability and prevent translocation of bacterial lipopolysaccharides (LPS) into systemic circulation [58]. Fermentation-enhanced phenolic content supports barrier integrity and helps prevent oxidative stress in the intestinal mucosa. This may contribute to intestinal homeostasis and reduce the risk of inflammatory bowel disease and metabolic endotoxemia [63]. 

Short-chain fatty acid (SCFA) metabolites suppress pro-inflammatory cytokine production (TNF-α, IL-1β, IL-6). Anti-inflammatory mediators such as IL-10 are also stimulated, contributing to cytokine modulation in colonic tissues. Production of short-chain fatty acids (SCFAs) by commensal bacteria may further support intestinal homeostasis and redox-sensitive immune regulation [58].

Gut microbiota-mediated biotransformation offers a plausible link between dietary antioxidants and cancer prevention through effects on bioavailability, inflammation, barrier function, and redox signaling [60].

Most of the available evidence remains mechanistic or translational, and direct human evidence linking specific microbial metabolites to cancer prevention is still limited [61].

At present, this field is supported more strongly by mechanistic and bioavailability data than by prevention-specific clinical outcomes [63].

## 9. Dietary Patterns and Translation to Cancer Prevention 

This section highlights representative large-scale cohort studies and meta-analyses selected for their methodological relevance, sample size, and translational value. The EPIC cohort and recent meta-analyses were prioritized because they provide large population samples, harmonized dietary-pattern assessments, clinically interpretable risk estimates, and high translational relevance for cancer prevention [11,41]. This section is not intended to provide an exhaustive systematic overview of all available clinical studies on dietary patterns and cancer prevention.

### 9.1. Mediterranean and Plant-Forward Dietary Patterns

Accumulating evidence from large-scale prospective cohorts indicates that adherence to Mediterranean and plant-forward dietary patterns is associated with modest but consistent reductions in cancer incidence and mortality. In the European Prospective Investigation into Cancer and Nutrition (EPIC) cohort of approximately 520,000 participants, followed for a median of 8.7 years, each two-point increment in Mediterranean diet score was associated with a 4% reduction in overall cancer risk (HR = 0.96, 95% CI 0.95–0.98), with similar effects observed in men and women [41]. Population-level modeling suggested that 4.7% of cancers in men and 2.4% in women could be avoided if adherence shifted to the highest category. Meta-analytic syntheses corroborate these findings, reporting a 13% reduction in cancer mortality among the general population (RR = 0.87, 95% CI 0.82–0.92) and a 25% reduction in all-cause mortality among cancer survivors (RR = 0.75, 95% CI 0.66–0.86) with the highest Mediterranean diet adherence [11].

Site-specific analyses reveal heterogeneous effects across cancer types. The strongest inverse associations emerge for gastrointestinal malignancies, including colorectal cancer (RR = 0.83, 95% CI 0.76–0.90), gastric cancer (RR = 0.70, 95% CI 0.61–0.80), and liver cancer (RR = 0.64, 95% CI 0.54–0.75), alongside head and neck cancers (RR = 0.56, 95% CI 0.44–0.72) [11]. Plant-based dietary patterns demonstrate comparable protective effects, with overall plant-based diet indices associated with 12% lower cancer mortality (RR = 0.88, 95% CI 0.79–0.98) and healthy plant-based indices showing 9% reductions (RR = 0.91, 95% CI 0.83–0.99), whereas unhealthy plant-based patterns characterized by refined grains and added sugars show null or adverse associations [83].

Several methodological limitations constrain causal inference. The proliferation of distinct Mediterranean diet scoring systems introduces substantial heterogeneity, with median intake thresholds varying markedly across populations [11]. The geographic concentration of evidence in European and North American populations limits generalizability to Asian and other populations with distinct dietary patterns and genetic backgrounds. Additional limitations of nutritional epidemiology include residual confounding, measurement error in dietary assessment, healthy-user bias, and potential reverse causation, which should be considered when interpreting observational associations between dietary patterns and cancer outcomes.

### 9.2. Whole Foods Versus Supplements: Risk Stratification

The health benefits associated with Mediterranean and plant-forward dietary patterns appear to arise from synergistic interactions between various bioactive components rather than the effects of singular nutrients. Component analyses from the EPIC cohort demonstrate that no single food group predominates; sensitivity analyses eliminating individual components sequentially, including alcohol, failed to identify a dominant component. Modest independent associations emerged for fruits and nuts (HR = 0.98 per 200 g), vegetables (HR = 0.97 per 145 g), cereals (HR = 0.97 per 110 g), and unsaturated-to-saturated lipid ratios (HR = 0.98 per 0.5-unit increment), suggesting additive rather than singular effects [41].

Baseline diet quality is an important determinant of cancer outcomes. Healthy plant-based patterns emphasizing whole grains, vegetables, and legumes reduce the risk of ovarian cancer (OR = 0.67, 95% CI 0.53–0.84), whereas unhealthy habits favoring refined carbohydrates and added sugars increase the risk (OR = 1.78, 95% CI 1.40–2.28) [84]. Occupational exposures were not systematically examined as effect modifiers in the reviewed literature, representing a gap in risk stratification frameworks.

To better illustrate the distinction between antioxidant-rich dietary patterns and isolated antioxidant supplementation, Table 2 summarizes the main conceptual and translational differences between these forms of antioxidant exposure in the context of cancer prevention.

This comparison highlights that the preventive effects of antioxidants are more consistently supported when they are consumed as part of whole-food dietary patterns rather than as isolated high-dose supplements.

## 10. Research Gaps and Methodological Considerations

Several critical research gaps limit the current understanding of dietary antioxidants in cancer prevention. We identified three main gaps in the current evidence:

1. The role of mitophagy in mediating the chemoprotective effects of dietary antioxidants is still insufficiently understood.
2. Experimental evidence supporting mitohormesis as a mechanism of dietary antioxidants remains limited.
3. The influence of gut microbiota metabolism on the bioactivity of dietary antioxidants is not fully clarified.

Methodological considerations for future research include the need for physiologically relevant doses and delivery methods, adequate study durations to capture long-term adaptive responses, improved dietary exposure assessment, microbiota and metabolomics readouts, incorporation of mechanistic biomarkers alongside clinical endpoints, and attention to food-matrix effects. Combinatorial approaches testing whole dietary patterns or synergistic combinations of bioactive compounds may prove more successful than single-compound interventions.

## 11. Discussion

The role of oxidative stress in cancer prevention is more complex than the traditional view [16]. Reactive oxygen species can promote either physiological adaptation or cellular damage, depending on the dose, timing, localization, and metabolic context [22]. This complexity helps explain why antioxidant strategies have produced mixed results across different stages of carcinogenesis [19].

The contrast between whole-food dietary patterns and isolated antioxidant supplementation further underscores this difference [11]. Diets rich in fruits, vegetables, legumes, whole grains, and other plant-derived foods are generally associated with a lower risk of cancer [11]. High-dose supplementation with isolated antioxidants has not consistently shown protective effects [12]; in some trials, isolated antioxidant supplements were associated with harm [39]. The findings of ATBC, CARET, and SELECT suggest that antioxidant supplementation cannot reproduce the complex biological effects of a whole diet [12]. This reflects the importance of food-matrix effects, synergistic interactions between bioactive compounds, and differences in baseline redox and metabolic status [69]. Current evidence suggests that the most plausible cancer-preventive effects are derived from antioxidant-rich dietary patterns and whole-food matrices, particularly those rich in polyphenols, carotenoids from foods, organosulfur compounds, and vitamin-containing plant foods [11]. High-dose isolated antioxidant supplementation may not be broadly protective [12].

To clarify why antioxidant studies have produced heterogeneous findings, Table 3 summarizes the main sources of inconsistency that may influence biological and clinical outcomes.

These factors suggest that apparently conflicting results across antioxidant studies may reflect differences in exposure type, host context, and biological timing, rather than a single uniform effect.

Another important insight from the current literature is that oxidative stress is closely integrated with inflammation, mitochondrial quality control, nutrient sensing, and gut microbiota metabolism [23]. Pathways such as NRF2-KEAP1, AMP-activated protein kinase–mechanistic target of rapamycin signaling, nuclear factor kappa B activation, autophagy, mitophagy, and inflammasome regulation do not operate in isolation [44]. Rather, they form an interconnected network that determines whether oxidative signals promote adaptation or contribute to disease [22]. Dietary antioxidants appear to act mainly by modulating this network, instead of simply scavenging reactive oxygen species [85].

### Future Directions: Biomarker-Guided and Mechanism-Based Prevention Trials

Building on the methodological gaps outlined above, future prevention studies should integrate mechanism-based trial design, population stratification, biomarker panels, dietary exposure assessment, microbiota and metabolomics readouts, and clinically meaningful cancer-related endpoints.

These observations have practical implications for cancer prevention. From a mechanistic perspective, preserving physiological redox signaling may be more biologically plausible than indiscriminate suppression of oxidative processes [16]. At the same time, the field still faces important limitations. Mitochondrial quality-control mechanisms, including autophagy and mitophagy, help limit the accumulation of damaged mitochondria and oxidative stress [33]. However, the direct relevance of these pathways to human cancer prevention remains limited and largely inferential [53]. Regular consumption of minimally processed plant foods appears more relevant to population-level prevention than routine use of high-dose antioxidant supplements [11]. High-dose isolated antioxidant supplementation has not consistently shown protective effects in cancer prevention trials [12]. Supplementation may still be considered in selected settings, such as documented deficiency states, but it should not be assumed to be broadly protective [36].

Individual responses to dietary antioxidants are shaped by baseline diet quality, metabolic status, inflammation, gut microbiota composition, and possibly occupational or environmental exposures [61]. These variables are rarely integrated into current prevention models [61]. A more precise understanding of redox biology may therefore require biomarker-guided approaches to identify when antioxidant interventions are beneficial, neutral, or potentially harmful [35].

Future progress in this area may depend on a better understanding of who is most likely to benefit from antioxidant-based interventions [35].

Relevant factors may include baseline redox status, inflammatory profile, metabolic health, antioxidant deficiency, obesity, insulin resistance, smoking exposure, and gut microbiota composition [35,61].

These variables may help explain why antioxidant interventions appear beneficial in some settings, neutral in others, and potentially harmful in selected cases [35,61].

For now, however, biomarker-guided redox nutrition should be regarded as a research priority rather than an established clinical strategy for cancer prevention [35].

Based on the evidence discussed in this review, candidate measurable biomarkers may include markers of oxidative damage and antioxidant defense, antioxidant nutritional status, inflammatory and metabolic profile, gut microbiota-derived metabolites, and dietary or supplement exposure context. These biomarkers remain investigational in the context of cancer prevention.

Based on the evidence discussed in this review, candidate measurable biomarkers may include markers of oxidative damage and antioxidant defense, antioxidant nutritional status, inflammatory and metabolic profile, gut microbiota-derived metabolites, and dietary or supplement exposure context. These biomarkers remain investigational in the context of cancer prevention. Table 4 summarizes candidate measurable biomarkers for future biomarker-guided redox nutrition studies in cancer prevention.

These biomarkers should be interpreted as investigational tools for future stratified prevention studies, not as established clinical criteria for antioxidant prescription in cancer prevention.

## 12. Conclusions and Future Directions

Dietary antioxidants should be understood within the broader context of redox homeostasis; factors such as dose, source, metabolic status, and disease stage influence their effects. ROS exert their effects through interactions with endogenous antioxidant systems, inflammatory pathways, and cellular metabolism.

The association between plant-rich dietary patterns and reduced cancer risk contrasts with the inconsistent outcomes of high-dose antioxidant supplementation. The effects of dietary antioxidants depend on biological context. These findings support a shift from isolated nutrient-based approaches toward whole-diet strategies. Current evidence appears to support antioxidant-rich whole-food patterns more consistently than isolated high-dose antioxidant supplements in the context of cancer prevention. These dietary patterns may provide polyphenols, food-derived carotenoids, organosulfur compounds from cruciferous and allium vegetables, and antioxidant vitamins within complex food matrices.

In conditions such as obesity and insulin resistance, redox signaling intersects with processes including mitochondrial function and inflammasome activation. The gut microbiota transforms dietary compounds into bioactive metabolites. This mechanism may explain the inter-individual variability in response.

Future research should prioritize the identification of reliable biomarkers of redox status, clarification of dose–response relationships, and stratification of populations most likely to benefit from specific interventions. Mechanism-based and personalized approaches can be essential for translating redox modulation into effective cancer prevention strategies.

In conclusion, it is essential to maintain redox homeostasis in a way that preserves physiological signaling while limiting chronic damage.

## Figures and Tables

**Table 1:** Major redox-sensitive molecular pathways and representative exposure contexts in cancer prevention.

Molecular Pathway/Mechanism	Main Role in Redox Regulation and Cancer Prevention	Representative Exposure Context/Examples	Relevance to Cancer Prevention/Key Caveat	Evidence Type/Directness to Prevention	Strength of Support	References
Redox hormesis/mitohormesis	Low-to-moderate ROS levels activate adaptive stress responses and enhance cellular resilience. Excessive antioxidant suppression may interfere with these beneficial signals.	Whole-food antioxidant-rich dietary patterns and food-matrix exposure; exercise-related endogenous adaptive responses; mitochondrial stress-responsive dietary bioactives.	Explains why mild oxidative signaling may support adaptive protection, whereas indiscriminate antioxidant supplementation may be detrimental.	Mechanistic; indirect human relevance.	High conceptual and mechanistic plausibility; limited direct human prevention evidence.	[19,42,43]
Nrf2-Keap1-ARE axis	Activates antioxidant, cytoprotective, and phase II detoxification pathways; supports redox homeostasis during early carcinogenesis.	Food-derived phytochemicals within plant foods, including cruciferous-vegetable-derived sulforaphane and polyphenol-rich foods containing curcumin, resveratrol, and quercetin; selected compounds may also be studied as isolated supplements or pharmacological Nrf2-modulating agents.	May help limit oxidative damage and carcinogen-induced cellular injury in early stages; persistent activation in established tumors may also support tumor adaptation.	Mechanistic/preclinical; stage-dependent relevance.	High mechanistic and preclinical support; stage-dependent relevance; limited direct human prevention evidence.	[34,45,46]
AMPK-mTOR-IGF-1 signaling	Integrates energy sensing, oxidative stress adaptation, mitochondrial metabolism, and anabolic signaling.	Polyphenol-rich food exposure, including grapes/berries, tea-derived catechins such as EGCG, and curcumin-containing dietary sources; isolated polyphenol supplements have also been used in mechanistic or translational studies.	Supports metabolic resilience and redox balance; however, effects are context-dependent and may differ between prevention settings and established tumors.	Mechanistic/metabolic; limited direct prevention evidence.	Moderate mechanistic and translational support; limited direct human prevention evidence.	[47,48,49]
NF-κB redox-sensitive inflammatory signaling	Links ROS excess to inflammatory gene expression, cytokine production, and chronic inflammatory signaling.	Anti-inflammatory food-derived polyphenol exposure, including curcumin-containing foods, quercetin-rich fruits and vegetables, resveratrol-containing foods, and tea-derived EGCG; isolated compounds are mainly relevant to mechanistic studies.	Relevant to chronic inflammation and early carcinogenic change; direct prevention evidence in humans remains limited.	Mechanistic/translational; indirect prevention relevance.	High mechanistic plausibility; moderate translational support; indirect prevention relevance.	[23,50,51,52]
Autophagy and mitophagy/mitochondrial quality control	Removes damaged ROS-producing mitochondria and preserves mitochondrial integrity through redox quality-control mechanisms.	Food-derived polyphenol exposure and dietary bioactives associated with mitochondrial quality control; examples include curcumin, quercetin, resveratrol, and tea catechins, mostly supported by mechanistic or preclinical studies.	May limit oxidative damage and mitochondrial dysfunction in early carcinogenesis; in established tumors, these pathways may also support survival under stress.	Mechanistic/preclinical; stage-dependent relevance.	High mechanistic and preclinical support; stage-dependent relevance; limited prevention-specific human evidence.	[33,53,54,55]
NLRP3 inflammasome and ROS crosstalk	Connects mitochondrial ROS, inflammasome activation, IL-1β/IL-18 signaling, and metabolic inflammation.	Food-derived polyphenols and antioxidant nutrients, including curcumin, quercetin, resveratrol, and vitamin-C-rich foods; high-dose vitamin C represents a pharmacological exposure and should be interpreted separately from dietary intake.	May contribute to prevention by limiting chronic redox-driven inflammation; effects remain context- and tissue-dependent.	Mechanistic/translational; context-dependent relevance.	Moderate mechanistic and translational support; context-dependent relevance; limited direct human prevention evidence.	[28,29,30,33,56,57]
Gut microbiota-mediated biotransformation of dietary antioxidants	Converts poorly absorbed parent compounds into bioactive metabolites that influence redox signaling, inflammation, and barrier integrity.	Polyphenol-rich foods and microbiota-dependent metabolites, including quercetin-derived phenolic acids, daidzein-derived equol, ellagitannin-derived urolithins, catechin-derived metabolites, and resveratrol-derived dihydroresveratrol.	Provides a plausible mechanistic link to cancer prevention through bioavailability and redox signaling; direct human prevention evidence remains limited.	Mechanistic/bioavailability; limited human prevention evidence.	Moderate bioavailability and mechanistic support; limited prevention-specific clinical evidence.	[58,59,60,61,62,63]

Note 1: “Strength of support” represents a qualitative narrative appraisal, not a formal GRADE assessment. The grading was based on the predominant evidence type, consistency of mechanistic findings, translational relevance, directness to cancer prevention, and availability of human prevention-oriented evidence. “High mechanistic support” indicates consistent mechanistic and/or preclinical evidence, but not necessarily direct human prevention evidence. “Moderate translational support” indicates biologically plausible evidence supported by mechanistic and selected translational or observational data. “Limited direct human prevention evidence” indicates that prevention-specific human intervention data remain scarce or indirect. Note 2: The “Representative exposure context/examples” column distinguishes food-derived exposure and whole-food matrices from isolated oral supplements and pharmacological redox-modulating interventions where relevant. This distinction is intended to avoid implying that all listed compounds have equivalent biological effects when consumed as part of foods, administered as supplements, or used at pharmacological doses.

**Table 2:** Conceptual comparison between whole-food antioxidant exposure and isolated antioxidant supplementation in cancer prevention.

Exposure Type	Biological Context	Typical Composition	Potential Advantages	Major Limitations/Caveats	Relevance to Cancer Prevention	References
Whole-food dietary patterns	Antioxidants are consumed within a complex food matrix and in combination with other nutrients and bioactive compounds	Fruits, vegetables, legumes, whole grains, nuts, olive oil, herbs, and other minimally processed plant foods	Provide synergistic interactions between polyphenols, carotenoids, vitamins, minerals, and fiber; support gut microbiota metabolism; associated with better overall dietary quality	Effects are difficult to attribute to a single compound; variability in dietary scoring systems and adherence; residual confounding in observational studies	Most consistently associated with lower cancer risk and mortality in epidemiological studies	[11,41,69,83,84]
Food-derived antioxidant exposure	Specific antioxidant compounds are consumed as part of foods rather than in isolated form	Polyphenols, carotenoids, organosulfur compounds, and vitamins C and E naturally present in foods	Greater physiological relevance; influenced by food matrix, digestion, and microbiota-derived biotransformation; may better preserve adaptive redox signaling	Bioavailability varies by food source, processing, and microbiota composition; dose is less standardized	Likely contributes to the protective effects of antioxidant-rich dietary patterns	[58,59,60,61,62,69]
Isolated oral antioxidant supplements	Selected antioxidants are administered outside the natural food matrix, often at relatively high doses	β-carotene, vitamin E, vitamin C, selenium, and single-compound polyphenol supplements	Easy to standardize; convenient administration; useful in deficiency states or targeted settings	May not reproduce food-matrix effects or compound synergy; effects depend on dose, baseline redox status, smoking status, and metabolic context; may be neutral or harmful in some populations	Evidence is inconsistent; some trials reported no benefit or increased cancer risk	[12,38,39,73]
Pharmacological antioxidant or redox-modulating interventions	Antioxidant-related compounds are used at pharmacological doses with therapeutic or experimental intent	High-dose intravenous vitamin C, Nrf2-targeting compounds, and pathway-specific redox modulators	May exert stronger and more targeted biological effects; useful for mechanistic exploration and possibly selected therapeutic settings	Different from nutritional prevention; pharmacological effects may include pro-oxidant activity; limited relevance to general-population prevention	Relevant mainly for mechanistic or therapeutic research, not for routine dietary cancer prevention	[26,36,45]

**Table 3:** Sources of inconsistency across antioxidant studies in cancer prevention.

Source of Inconsistency	Relevance	Examples/Implications	References
Dose	Antioxidants may exert different biological effects at physiological versus high doses.	Low dietary exposure may support adaptive redox balance, whereas high-dose supplementation may disrupt physiological ROS signaling or produce unexpected effects.	[12,36]
Chemical form and exposure matrix	The biological effects of antioxidants may differ when compounds are consumed as part of foods compared with isolated supplemental forms.	Food-derived antioxidants are delivered within complex matrices, whereas isolated supplements may differ in absorption, metabolism, and biological context.	[11,69]
Whole foods versus isolated supplements	Food matrices provide fiber, micronutrients, and multiple bioactive compounds that may act synergistically.	Antioxidant-rich dietary patterns are more consistently associated with lower cancer risk than isolated oral supplements.	[11,41,69]
Baseline nutritional and redox status	The effects of antioxidant interventions may depend on whether deficiency, oxidative burden, or altered redox balance is already present.	Supplementation may be better supported in deficiency states than in individuals with adequate baseline antioxidant status.	[35,36]
Metabolic and environmental context	Host context may influence the biological effects of antioxidant interventions.	Smoking-related oxidative burden may partially explain harmful findings in β-carotene trials, while obesity-associated low-grade inflammation may modify cancer-related inflammatory signaling.	[38,39,40,57]
Biological timing and disease stage	The same pathway may be protective in early carcinogenesis but supportive of survival in established tumors.	Antioxidant signaling may help prevent oxidative damage early yet favor tumor adaptation or progression in established cancer.	[19,26,74]
Gut microbiota and bioavailability	Inter-individual differences in microbial metabolism may alter the generation of bioactive metabolites.	Food-derived polyphenols may be transformed into metabolites with distinct biological effects, contributing to variable responses across individuals.	[60,61,62]
Population risk profile, cancer site, and endpoint definition	Trial results may differ according to cancer site, smoking status, baseline nutritional status, follow-up duration, and endpoint selection.	β-carotene trials in smoker-enriched populations reported harmful lung-cancer-related outcomes, whereas SELECT primarily addressed the risk of prostate cancer. Differences in follow-up duration and endpoints may contribute to heterogeneous findings.	[38,39,40]

**Table 4:** Candidate measurable biomarkers for future biomarker-guided redox nutrition studies in cancer prevention.

Biomarker Category	Candidate Measurable Biomarkers	Biological Interpretation	Potential Stratification Use	Main Limitation	References
Oxidative damage/redox status	8-oxo-dG; antioxidant enzyme activity	Oxidative DNA damage and antioxidant defense response	Identify individuals with increased oxidative burden	No standardized cancer-prevention thresholds	[18,35]
Antioxidant nutritional status	Plasma vitamin C; serum/plasma α-tocopherol; carotenoids; selenium; zinc; DAI	Antioxidant reserve, intake, or deficiency state	Identify deficiency or low antioxidant status	Influenced by diet, supplements, absorption, and assay method	[35,36,68,69,73]
Inflammatory/metabolic profile	hs-CRP; IL-6; TNF-α; IL-1β; IL-18; BMI; waist circumference; fasting glucose; HbA1c	Low-grade inflammation, inflammasome activity, obesity, and dysglycemia	Identify inflammatory or metabolically high-risk phenotypes	Non-specific; influenced by comorbidities and medications	[23,29,31,50,57,80]
Gut microbiota/metabolites	Microbiota composition; SCFAs; urolithins; equol; phenolic acids	Microbial biotransformation capacity and inter-individual variability	Predict response to polyphenol-rich dietary patterns	Requires microbiome/metabolomics methods	[58,59,60,61,62,63]
Dietary and exposure context	Mediterranean diet score; plant-based diet quality; supplement use; smoking status	Food-matrix exposure versus isolated supplementation and oxidative burden	Distinguish whole-food exposure from high-dose supplement context	Self-report bias and residual confounding	[11,12,38,39,40,41,69,73,83,84]

## Data Availability

No new data were created in this study.

## Abbreviations

The following abbreviations are used in this manuscript:

Akt	Protein Kinase B
AMPK	AMP-Activated Protein Kinase
ARE	Antioxidant Response Element
ATBC	Alpha-Tocopherol, Beta-Carotene Cancer Prevention Study
ATP	Adenosine Triphosphate
BNIP3	BCL2 Interacting Protein 3
CARET	Beta-Carotene and Retinol Efficacy Trial
cGAS	Cyclic GMP–AMP Synthase
CI	Confidence Interval
DADS	Diallyl Disulfide
DAI	Dietary Antioxidant Index
DATS	Diallyl Trisulfide
DNMTs	DNA Methyltransferases
EGCG	Epigallocatechin Gallate
EMT	Epithelial-to-Mesenchymal Transition
EPIC	European Prospective Investigation into Cancer and Nutrition
ERK	Extracellular Signal-Regulated Kinase
FoxO	Forkhead Box O Transcription Factors
HIF-1α	Hypoxia-Inducible Factor 1-alpha
HO-1	Heme Oxygenase 1
HR	Hazard Ratio
IGF-1	Insulin-like Growth Factor 1
IKK	IκB Kinase
IκB	Inhibitor of κ
IL	Interleukin
IL-1β	Interleukin 1 Beta
IL-6	Interleukin 6
IL-10	Interleukin 10
KEAP1	Kelch-like ECH-Associated Protein 1
LPS	Lipopolysaccharide
MAPK	Mitogen-Activated Protein Kinase
MDSCs	Myeloid-Derived Suppressor Cells
mPTP	Mitochondrial Permeability Transition Pore
mTOR	Mechanistic Target of Rapamycin
mTORC1	Mechanistic Target of Rapamycin Complex 1
mtROS	Mitochondrial Reactive Oxygen Species
NAD^+^	Nicotinamide Adenine Dinucleotide
NFE2L2	Nuclear Factor Erythroid 2–Related Factor 2
NF-κB	Nuclear Factor Kappa B
NLR	NOD-Like Receptor
NLRP3	NOD-, LRR- and Pyrin Domain-Containing Protein 3
NOX	NADPH Oxidase
Nrf2	Nuclear Factor Erythroid 2–Related Factor 2
OR	Odds Ratio
PGC-1α	Peroxisome Proliferator-Activated Receptor Gamma Coactivator 1-alpha
PINK1	PTEN-Induced Kinase 1
PTEN	Phosphatase and Tensin Homolog
PTP1B	Protein Tyrosine Phosphatase 1B
ROS	Reactive Oxygen Species
RR	Relative Risk
SCFA	Short-Chain Fatty Acid
SELECT	Selenium and Vitamin E Cancer Prevention Trial
SFN	Sulforaphane
SIRT1	Sirtuin 1
SOD	Superoxide Dismutase
STING	Stimulator of Interferon Genes
TGF-β	Transforming Growth Factor Beta
TLR4	Toll-Like Receptor 4
TNF-α	Tumor Necrosis Factor Alpha
ULK1	Unc-51 Like Autophagy Activating Kinase 1
VDAC	Voltage-Dependent Anion Channel

## Appendix A

**Table A1:** Search strategy used to identify relevant literature for the structured narrative review.

Source/Tool	Search Approach	Search Concepts	Filters/Notes
PubMed	MeSH + title/abstract; main search plus pathway-specific searches	(“Antioxidants”[Mesh] OR “Phytochemicals”[Mesh] OR “Polyphenols”[Mesh]OR “Carotenoids”[Mesh] OR antioxidant*[tiab]OR “dietary antioxidant*”[tiab] OR phytochemical*[tiab] OR polyphenol*[tiab] OR carotenoid*[tiab] OR sulforaphane[tiab] OR curcumin[tiab] OR resveratrol[tiab] OR quercetin[tiab] OR EGCG[tiab]) AND (“Oxidative Stress”[Mesh] OR “Reactive Oxygen Species”[Mesh] OR “redox signaling”[tiab] OR “redox homeostasis”[tiab] OR “oxidative stress”[tiab] OR “reactive oxygen species”[tiab]) AND (“Neoplasms/prevention and control”[Mesh] OR “Carcinogenesis”[Mesh] OR “cancer prevention”[tiab] OR “early carcinogenesis”[tiab] OR carcinogenesis[tiab]) AND (“NF-E2-Related Factor 2”[Mesh] OR “NF-kappa B”[Mesh] OR “AMP-Activated Protein Kinases”[Mesh] OR “TOR Serine-Threonine Kinases”[Mesh] OR “Inflammasomes”[Mesh] OR “Autophagy”[Mesh] OR “Mitophagy”[Mesh] OR “Gastrointestinal Microbiome”[Mesh] OR Nrf2[tiab] OR “Keap1”[tiab] OR “AMPK”[tiab] OR “mTOR”[tiab] OR “NLRP3”[tiab] OR “Inflammasome*”[tiab] OR “autophagy”[tiab] OR “mitophagy”[tiab] OR “gut microbiota”[tiab])	English; 2021–2026; humans and other animals; reviews, systematic reviews, meta-analyses, clinical/observational studies
Scopus	TITLE-ABS-KEY	((“dietary antioxidants” OR phytochemical* OR polyphenol* OR carotenoid* OR sulforaphane OR curcumin OR resveratrol OR quercetin OR EGCG) AND (“redox signaling” OR “redox homeostasis” OR “oxidative stress”) AND (“cancer prevention” OR “early carcinogenesis” OR carcinogenesis) AND (Nrf2 OR Keap1 OR AMPK OR mTOR OR “NF-kB” OR “NF-κB” OR NLRP3 OR inflammasome OR autophagy OR mitophagy OR “gut microbiota”))	English; 2021–2026; articles/reviews; journals
Web of Science Collection	TS=	(Antioxidants OR Phytochemicals OR Polyphenols OR Carotenoids OR antioxidant* OR “dietary antioxidant*” OR phytochemical* OR polyphenol* OR carotenoid* OR sulforaphane OR curcumin OR resveratrol OR quercetin OR EGCG) AND (“Oxidative Stress” OR “Reactive Oxygen Species” OR “redox signaling” OR “redox homeostasis” OR “oxidative stress” OR “reactive oxygen species”) AND (“cancer prevention” OR “early carcinogenesis” OR carcinogenesis) AND (“NF-E2-Related Factor 2” OR “NF-kappa B” OR “AMP-Activated Protein Kinases” OR “TOR Serine-Threonine Kinases” OR Inflammasomes OR Autophagy OR Mitophagy OR “Gastrointestinal Microbiome” OR Nrf2 OR Keap1 OR AMPK OR mTOR OR NLRP3 OR inflammasome* OR autophagy OR mitophagy OR “gut microbiota”)	English; 2021–2026; articles/reviews

The asterisk (*) was used as a truncation/wildcard operator to retrieve word variants; TS= was used as the Web of Science Topic search field tag.

**Table A2:** Literature selection criteria for the structured narrative review.

Inclusion	Exclusion
Studies evaluating dietary antioxidants, phytochemicals, polyphenols, carotenoids, organosulfur compounds, antioxidant vitamins, or antioxidant-rich dietary patterns	Studies not related to dietary antioxidants, phytochemicals, oxidative stress, redox biology, or cancer prevention
Human, animal, and experimental studies relevant to cancer prevention, early carcinogenesis, cancer biology, redox homeostasis, metabolic inflammation, mitochondrial quality control, or gut microbiota-mediated metabolism	Studies focused exclusively on established cancer treatment without relevance to prevention, early carcinogenesis, redox mechanisms, or dietary modulation
Studies reporting outcomes or mechanisms related to oxidative stress, reactive oxygen species, redox signaling, antioxidant defense, inflammatory pathways, mitochondrial function, autophagy, mitophagy, or metabolic alterations associated with carcinogenesis	Studies without mechanistic, metabolic, inflammatory, mitochondrial, microbiota-related, or cancer-prevention relevance
Studies investigating redox-sensitive molecular pathways, including Nrf2-Keap1, NF-κB, AMPK-mTOR, inflammasomes, NLRP3, autophagy, mitophagy, and gut microbiota-mediated biotransformation	Studies focused only on pharmacologic oncology interventions without dietary, nutritional, antioxidant, phytochemical, or redox-related relevance
Clinical studies, observational studies, cohort studies, systematic reviews, meta-analyses, narrative reviews, and mechanistic/preclinical studies relevant to the review objective	Conference abstracts, editorials, opinion pieces, letters, and case reports without mechanistic discussion or original/relevant synthesis
Articles published between 2021 and 2026	Articles published outside the 2021–2026 interval, unless considered earlier seminal studies or landmark clinical/cohort evidence
Earlier seminal studies, major clinical trials, large cohort studies, and key mechanistic papers were included when directly relevant to the conceptual framework of the review	Older articles not considered seminal, landmark, or directly relevant to the review objective
Articles published in English	Non-English language publications
Studies with accessible abstract and sufficient methodological or mechanistic information for thematic interpretation	Studies with insufficient information to assess relevance to the review objective
